# Ophthalmic Complications of Dengue

**DOI:** 10.3201/eid1202.050274

**Published:** 2006-02

**Authors:** David P.L. Chan, Stephen C.B. Teoh, Colin S.H. Tan, Gerard K.M. Nah, Rajesh Rajagopalan, Manjunath K. Prabhakaragupta, Caroline K.L. Chee, Tock H. Lim, Kong Y. Goh, on behalf of The Eye Institute Dengue-Related Ophthalmic Complications Workgroup

**Affiliations:** * The Eye Institute at Tan Tock Seng Hospital, Republic of Singapore;; † Singapore National Eye Research Institute, Singapore;; ‡ The Eye Institute at National University Hospital, National University Hospital, Singapore

**Keywords:** Dengue hemorrhagic fever, retinopathy, maculopathy, vasculitis, inflammation, eye, ophthalmic complications, research

## Abstract

We report 13 cases of ophthalmic complications resulting from dengue infection in Singapore. We performed a retrospective analysis of a series of 13 patients with dengue fever who had visual impairment. Investigations included Humphrey automated visual field analyzer, Amsler charting, fundus fluorescein angiography, and optical coherence tomography. Twenty-two eyes of 13 patients were affected. The mean age of patients was 31.7 years. Visual acuity varied from 20/25 to counting fingers only. Twelve patients (92.3%) noted central vision impairment. Onset of visual impairment coincided with the nadir of serum thrombocytopenia. Ophthalmologic findings include macular edema and blot hemorrhages (10), cotton wool spots (1), retinal vasculitis (4), exudative retinal detachment (2), and anterior uveitis (1). All patients recovered visual acuity to 20/30 or better with residual central scotoma by 12 weeks. These new complications suggest a widening spectrum of ophthalmic complications in dengue infection.

## Abstract 2

A case series suggests that the spectrum of complications in dengue infection is widening.

Dengue fever (DF) is the most prevalent form of flavivirus infection in humans. Borne by the *Aedes* mosquito, the infection is endemic in the tropics and warm temperate regions of the world. The highest incidence occurs in Southeast Asia, India, and the American tropics. Worldwide cases of illness exceed100 million per year (*1**,**2*).

Dengue hemorrhagic fever (DHF) is a severe and potentially fatal form of the disease. Twenty-five thousand deaths are reported annually to the World Health Organization (WHO). The annual incidence now exceeds 500,000 cases annually and is still rising, despite environmental controls (*3*). DHF is strongly related to previous sensitization of heterologous dengue infection. Increasing endemicity and co-circulation of different serotypes is therefore necessary for the increase in incidence of DHF.

DF is characterized by an abrupt onset of fever after a 2- to 7-day incubation period, with temperatures reaching 41°C. Other symptoms include severe malaise, headaches, and retroorbital and lumbrosacral pain. Patients also experience respiratory symptoms (sore throat, rhinitis, and cough), nausea, anorexia, and altered taste sensation. A transient macular rash is often seen on day 1 to day 2 of illness. This rash disappears, but a second, maculopapular rash appears on days 3–6 of illness. The secondary rash coincides with defervescence and typically involves the trunks, limbs, and face; palms and soles are spared. Blood dyscrasias include thrombocytopenia and neutropenia (leukopenia). The illness is usually self-limiting with minimal systemic sequelae, but it may require prolonged convalescence lasting several weeks.

DHF is defined by WHO as DF associated with thrombocytopenia (<100 × 10^9^ cells/L) and hemoconcentration (hematocrit >20% above baseline). Its most severe form, dengue shock syndrome (DSS), is associated with hypotension, narrowing of pulse pressure (<20 mm Hg), and circulatory failure in 30% of cases. The early phase of DHF is indistinguishable from DF. The death rate for untreated DHF/DSS can be as high as 10%–15% in places where emergency supportive treatment with intravenous fluids and platelet replacement is not readily accessible (*4*).

Ophthalmic complications associated with DF and DHF have not been classically described. Within the ophthalmic community, this complication is being observed more frequently in recent times. However, only a few isolated case reports have been published (*5**–**13*). These reports attribute ocular complications to the transient thrombocytopenia and resulting bleeding diathesis. The course of the eye manifestations has also not been well-described. We report a series of 13 patients who had ophthalmic symptoms after DF or DHF, and describe the course, spectrum of manifestations, and prognosis and treatment of these new and emergent complications.

## Methods

We describe a retrospective observational case series of 13 patients who were seen at The Eye Institute (Singapore) over 6 months from September 2004 to February 2005. Patient follow-up varied from 2 weeks to 5 months. Diagnosis was made by a referring infectious disease physician on the basis of characteristic clinical signs and symptoms and confirmed on dengue polymerase chain reaction (PCR), dengue serology (immunoglobulin M [IgM] and IgG seroconversion), or both.

Real-time automated reverse transcriptase (RT)-PCR assay was conducted with the Dengue LC RealArt RT-PCR Kit on the Light Cycler (Roche Diagnostics, Mannheim, Germany) in patients with <5 days of fever. In patients with pyrexia in excess of 5 days, serologic studies were conducted with the PanBio (Sinnamon Park, Queensland, Australia) Dengue Duo IgM and IgG Rapid Strip Test. Classification of DF and DHF was made on the basis of WHO guidelines.

Patients were referred to The Eye Institute following complaints of visual symptoms. All patients had visual acuity measured with a Snellen acuity chart. All underwent a full slit-lamp anterior segment examination as well as dilated fundi examination with slit-lamp biomicroscopy. Upon clinical diagnosis, patients underwent further testing of visual fields (Humphrey automated visual field analyzer [HVF], Amsler charting, and fundal fluorescein angiography [FA]), and measurement of central macular thickness with optical coherence tomography (OCT3, Zeiss, Göttingen, Germany).

Patients were followed up by examination of serial platelet counts until at least 2 consecutive counts showed an upward trend. Retinal findings were documented with serial color fundal photography. Tests (HVF, FA, and OCT) were repeated based on clinical assessment of the patient's response and clinical signs of resolution.

## Results

### Demographics

**Figure A1:**
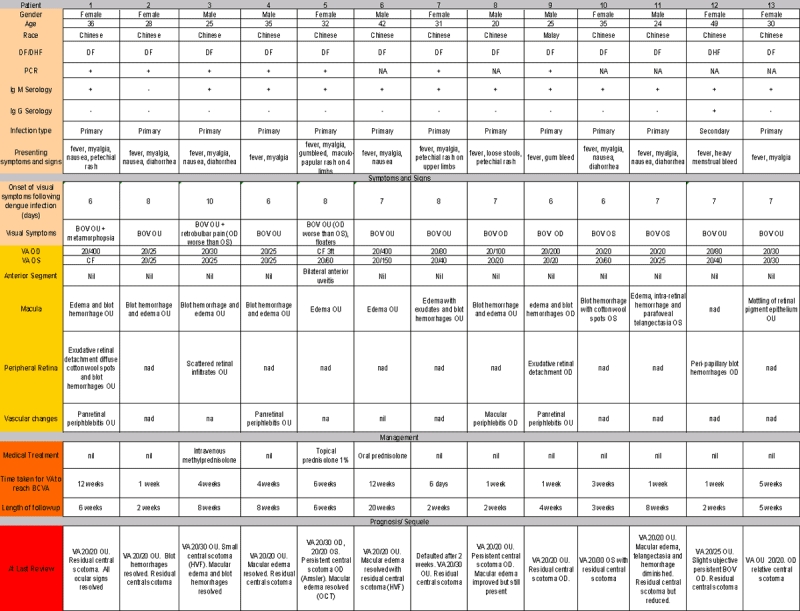
Summary of patient demographics, manifestations, clinical findings, management, and outcome. DF, dengue fever; DHF , dengue hemorrhagic fever; NA, not available; BOV, blurring of vision; VA, visual acuity (Snellen); OU, both eyes; OD, right eye; OS, left eye.

Thirteen patients (6 male, 7 female) with ophthalmic symptoms following DF were reviewed. Eleven patients were Singaporeans; the other 2 were Chinese nationals. All cases were contracted in Singapore, based on the absence of travel history 1 month before the illness. All but 1 patient (Malay) was of Chinese race. The ages ranged from 20 to 49 years (mean 31.7 ± 7.9 years, median 31 years) with no age differences between male and female patients. All but 1 patient were classified as having DF (Figure A1).

### Symptoms

**Figure 1:**
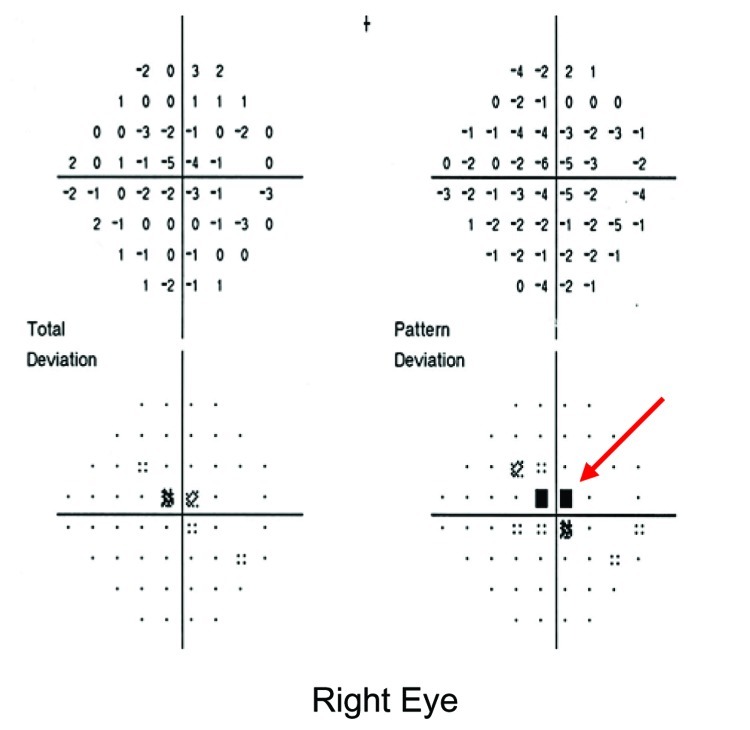
Humphrey visual fields of patient 9 at 1 week after onset of visual symptoms. Central scotoma of the right visual field is denoted as black squares (red arrow).

All patients complained of blurring of vision. Nine patients described bilateral visual symptoms in both eyes; 4 (30.7%) noted unilateral visual impairment. Twenty-two eyes from 13 patients were affected. Snellen visual acuity varied from 20/25 to counting fingers only (median 20/40). Seven eyes (31.8%) had vision of 20/100 or worse. Twelve (92.3%) patients described blurring associated with a loss of central vision (relative central scotoma) (Figure 1). This symptom was demonstrated on Amsler charting and automated HVF testing.

### Onset of Visual Symptoms

**Figure 2:**
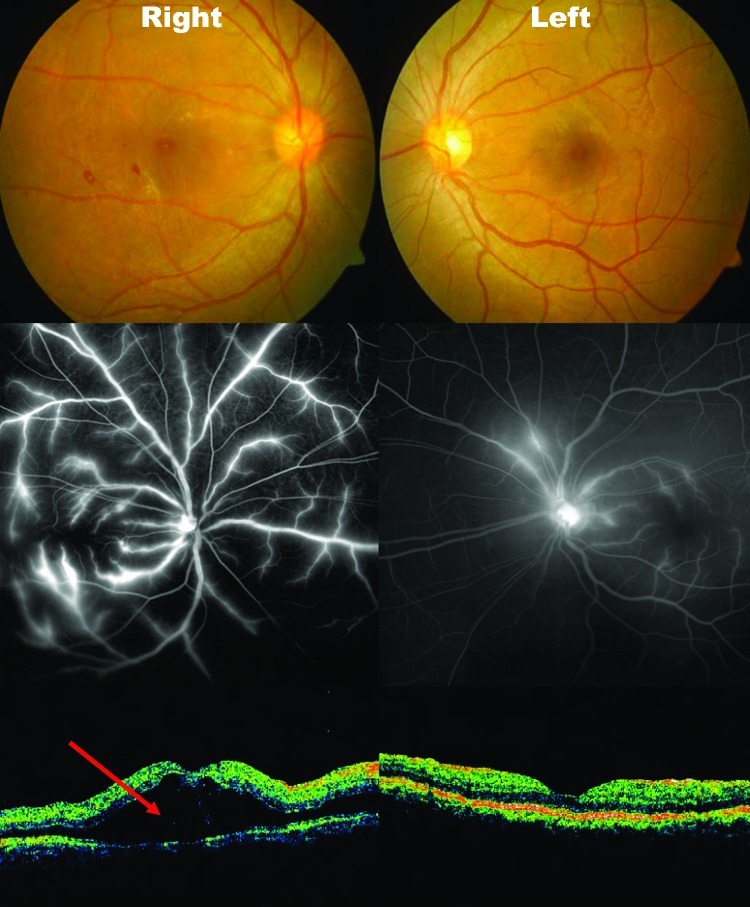
Trend of serial serum platelets after the onset of dengue virus infection.

The onset of visual symptoms closely correlated with the nadir of thrombocytopenia associated with DF. Of the 9 patients with available daily serial serum platelet measurements, all had visual symptoms within 1 day of their lowest platelet counts. Five (55.6%) patients complained of visual symptoms on the day of their nadir; 2 patients exhibited this symptom 1 day after their lowest count, and 2 patients had this symptom 1 day before their lowest count (mean 6.8 ± 0.8 days, median 7 days) (Figure 2).

### Signs

**Figure 3:**
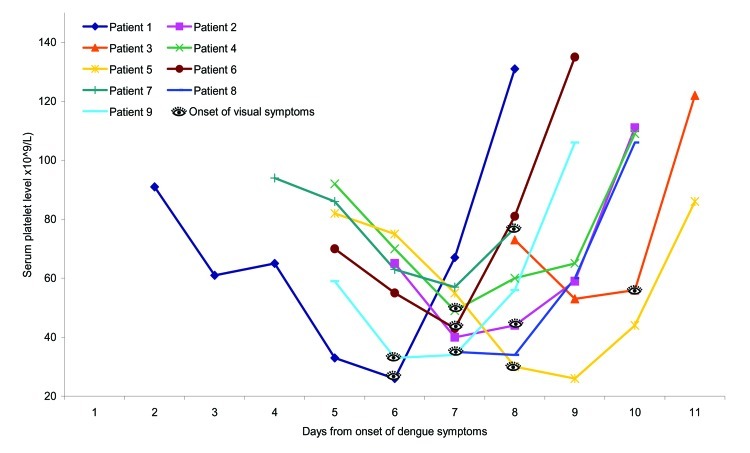
Fundal photos, fundal fluorescein angiography and optical coherence tomography (OCT) of patient 9. A) areas of blot hemorrhages temporal to the right fovea. B) bilateral dye leakage from the retinal veins, more severe on the right than left. C) OCT gives a 2-dimensional graphic representation of a cross-section of the macular region. The area marked with the red arrow marks the site of exudative retinal detachment. Both sides have marked retinal thickening (edema). Photo: Ken Thian.

The most common ophthalmic signs were found on the macular region of the retina (Figure 3). Macular edema was the most common pathology; it occurred in 10 (76.9%) patients. The second most common finding on ophthalmoscopy was macular hemorrhage (9 [69.2%] patients). Characteristically, these took the form of blot hemorrhages. These areas corresponded to the areas of visual scotoma experienced by the patients. Four cases of vasculitis occurred. One involved the macular vasculature, and 3 patients had panretinal vasculitis. Two cases with severe panretinal vasculitis were associated with exudative retinal detachment. Other less common fundus findings include perifoveal telangectasia and cotton wool spots, both at the macula and peripheral retina. Anterior segment findings were relatively uncommon in our series. Only 1 patient had associated anterior uveitis.

### Investigations

Diagnosis of dengue was based on a combination of clinical findings correlated to positive results from dengue serology, PCR, or both. Serial serum platelet measurements tracked the thrombocytopenic pattern. All but 4 patients had available serial serum platelet measurements. Three patients had their first platelet measurements only after 1 week of visual symptoms. One patient had been transfused before onset of visual symptoms, but even then, when examined, her platelet count was only 5.0 × 10^9^ cells/L. All but 1 patient was classified as having primary dengue. Patients with <5 days of dengue symptoms with a positive PCR but negative IgG serologic findings were deemed to have primary dengue. Alternatively, patients with dengue symptoms of >5 days' duration who had a positive IgG serologic finding were classified as having secondary dengue infection.

The mean platelet nadir at the time of onset of visual complaints was 42.8 ± 20.1 × 10^9^ cells/L (range 5–77 × 10^9^ cells/L, normal 160–390 × 10^9^ cells/L). Complete blood count showed that these findings corresponded to their peak hematocrit of 43.0 ± 4.3% and a leukopenia nadir of 2.4 ± 1.0 × 10^9^ cells/L (range 1.3–3.9 × 10^9^ cells/L, normal 4–10 × 10^9^ cells/L). All patients demonstrated central scotoma due to macular pathology by means of Amsler chart reading and automated visual field (HVF) testing. Fundal fluorescein angiography (FA) performed in 4 severe cases demonstrated extensive fluid leakage from retinal vessels corresponding to clinical observation of macular edema (Figure 3B) and peripheral vasculitis. This finding was corroborated on OCT (Figure 3C), which showed thickening of the macula.

### Management and Progress of Retinopathy

All but 2 patients were treated conservatively. For these 11 patients, clinical signs resolved spontaneously and rapidly after they recovered from thrombocytopenia (median 3 days). Two patients with extensive panretinal vasculitis and exudative detachment were treated with systemic steroids. One patient was given oral prednisolone at a dose of 1 mg/kg/day for 1 week; this dosage was tailed off slowly over 2 months. The other patient received 6 hourly doses of intravenous methylprednisolone 250 mg for 3 days, followed by oral prednisolone at 1 mg/kg/day for 1 week, tailed off over the next 2 months in a similar manner. None of the patients who had steroid treatment reported adverse effects after steroid treatment. Both patients demonstrated visual recovery with resolution of clinical signs after 1 month. One patient with bilateral anterior uveitis was treated with topical prednisolone 1%. The anterior uveitis resolved by day 7 with no subsequent relapse, and the medication was tapered off (Figure A1).

### Outcomes and Prognosis

One patient defaulted follow-up after 2 weeks because vision had returned to normal. The remaining 12 patients had a recovery period between 6 days to 3 months. Resolution of clinical signs was closely followed by improvement of their Snellen acuity back to pre-retinopathy levels. Nine patients (75%) achieved a best corrected visual acuity of 20/25 or better (mean 4.0 weeks). However, despite resolution of ocular signs, all reported residual mild central scotoma that was reflected on HVF as an area of subtle decrease in sensitivity in the central vision (Figure 1). This persisted even up to 3 months after complete systemic recovery.

## Discussion

Dengue is the most common mosquitoborne viral disease in humans. In recent years, it has become a major international public health concern. Globally, 2.5 billion people live in areas where dengue viruses can be transmitted (*4**,**14**–**16*). Over the past 25 years, the geographic spread of both the mosquito vectors and the viruses has led to the global resurgence of epidemic DF and emergence of DHF; with the development of hyperendemicity in many urban centers of the tropics. Though Southeast Asian in origin, this study would be relevant to clinicians across continents where dengue has taken a foothold.

The spectrum of ophthalmologic manifestations would lead one to conclude that several pathophysiologic processes are involved. The first and most obvious pathogenesis would be the thrombocytopenic state, with its resultant bleeding tendency, which gives rise to increased incidence of hemorrhage. These hemorrhages manifest as retinal blot hemorrhages in the macula and retinal periphery. We believe that the preponderance of cases found with complications located at the macula in our series may be due to the higher likelihood of awareness by the patient of visual impairment resulting from poor central vision. The incidence of dengue-related complications may be higher, given that some patients with changes occurring exclusively in the retinal periphery may not have any perceptible visual impairment. Macular edema and occult vascular changes with minimal functional disturbance may also be unreported by the patient. Clinically, these cases may even be missed on examination alone. Investigations such as fundus FA and OCT can help to detect these occult cases. These signs could also lend insight to the microvascular changes that may be occurring in the rest of the body. However, a hypocoagulable state alone would not account for the entire range of complications seen. The presence of periphlebitis, anterior uveitis, and macular edema indicate a hyperpermeable and inflammatory process. Parallels can also be drawn from the observation that the visual symptoms tend to occur and manifest at or close to the moment when the serum platelets and leukocytes levels reach their trough, while the disease is at its peak.

An hypothesis about the pathogenesis of DHF, though proven true in vivo, involves immune clearance by way of induction of cross-reactive T-cell memory, T-cell proliferation, and recognition of dengue viral antigens on infected monocytes by sensitized CD4+CD8– and CD4–CD8+ cytotoxic T cells. This results in the release of cytokines with vasoactive and procoagulant properties (interleukins, tumor necrosis factor, platelet-activating factor, and urokinase) (*17**,**18*). Vasoactive and inflammatory mediators cause capillary leakage, which may form the basis for macular edema and breakdown of the aqueous blood barrier, resulting in anterior uveitis and periphlebitis. In the series reported by Lim et al., ocular complications were mainly confined to the maculae (*5*). However, in our series the extent of involvement includes both the peripheral retina in the posterior segment and the anterior segment (anterior uveitis), which suggests a more widespread inflammatory process in the eye.

The onset of visual symptoms occurs on or close to the day of the lowest serum platelet level. Visual recovery, in the form of improvement of signs and symptoms, usually corresponds to improving platelet levels but may take several weeks to reach a steady state. Most patients report residual visual impairment in the form of central or paracentral scotoma.

The use of systemic steroids in 2 patients did not appear to aggravate the visual complications or the systemic dengue infection. This finding is supportive of an inflammatory or immune-mediated pathophysiology after acute dengue infections. Visual symptoms and visual acuity recovered in the same manner and speed as in patients with milder, untreated cases. However, like the other patients, both also described a persistent central scotoma despite normal functional Snellen visual acuities. However, we were not able to draw any statistical conclusions on the efficacy of treatment outcomes.

Our findings may have arisen as a result of an increase in incidence and awareness of DF in Singapore (*19*). However, we believe that these complications may constitute a change in the pathoimmunology of the disease. The increase in inflammatory response seen in recent DF patients may be due to a change in pathogenicity of the virus, although any viral mutation would be speculative at best with our current understanding of the disease. Hence, the identification of serotypes or viral RNA epitopes in future studies might identify particular serotype or combinations of serotypes, as in the case of secondary infections, of heterologous dengue serotypes that might be found to confer a higher risk of ocular and possibly systemic complications.

This case series describes the widest variety of ocular complications of dengue infection to date. Although the ophthalmic community has been reporting more of such cases in recent times, the number of cases in this series is still relatively small and represents a limitation to the results of this report. No attempt at randomization had been made with regards to treatment. Management was based on clinical judgment on the progress of pathologic features. However, we feel that the consistency of visual outcomes in these patients still reflects the course of dengue-related ophthalmic complications.

In conclusion, DF and DHF can cause ophthalmic symptoms that were not previously well-described in the medical literature. Blurring of vision typically coincides with the nadir of thrombocytopenia and occurs ≈1 week after onset of fever. Clinical features include retinal edema, blot hemorrhages, and vasculitis. Less common features include exudative retinal detachment, cotton wool spots, and anterior uveitis.

Prognosis is generally good as the disease is often self-limiting, resolving spontaneously even without treatment. However, patients may experience mild relative central scotoma that may persist for months. The use of steroids in treating this inflammatory eye condition is controversial. A randomized controlled trial is under way to evaluate the effect of systemic steroids on dengue retinopathy; results will be reported in due course.

With increasing epidemicity and co-circulation of multiple dengue serotypes, the occurrence of DF and DHF is set to rise. Similarly we expect to see an increase in this newly emergent facet of dengue ophthalmic morbidity. A heightened awareness of dengue-related ophthalmic complications among clinicians involved in the care of patients with dengue would facilitate prompt referral for ophthalmologic assessment and management.
